# Peer Review in *PLoS Medicine*

**DOI:** 10.1371/journal.pmed.0040058

**Published:** 2007-01-30

**Authors:** The PLoS Medicine Editors

## Abstract

The PLoS Medicine editors discuss a paper about the difficulties of identifying what makes a good peer reviewer, and the process of peer review in PLoS Medicine.

Skill in scientific peer review may be as ill defined and hard to impart as is “common sense” [1]. So say Michael Callaham and John Tercier, the authors of a research paper published in this month's issue of *PLoS Medicine*, which assessed 2,856 reviews of 306 experienced reviewers at one well-regarded specialty journal. The study concluded that the only significant positive predictors of review quality were reviewers who were less than 10 years out of training or those that worked in a teaching hospital environment.

These are somewhat alarming conclusions for us, as editors of a peer-reviewed journal, but not ones that are completely surprising. A previous study concluded that the only predictor of good performance of reviewers of a general medical journal was prior training in epidemiology or statistics [2] (an effect not found by Callaham and Tercier). A previous randomized controlled trial showed moreover that training has only a small and short-lived effect on reviewers' performance [3]. When these findings are taken alongside the scant objective evidence that peer review works at all in identifying even errors deliberately introduced by researchers to investigate peer review [4,5], let alone scientific fraud [e.g., 6,7], one might wonder why journals bother with peer review at all.

But, somehow peer review has come to be considered a badge of respectability among journals. Why this has happened is perhaps worth exploring. Peer review—the process of showing work to colleagues for their comments—is as old as research itself. But the formal process of peer review as undertaken by scientific journals is rather a new innovation. Until the middle of the 20th century most work published in journals was not peer reviewed but published at the discretion of the editors. On some occasions editors, as academics themselves, might have been considered peers of the authors, but on other occasions must have evaluated work well outside their area of expertise. If advice was sought it was primarily to confirm the intended rejection of papers that seemed bizarre, but arguably such a system allowed the publication of very innovative ideas too. Some have argued that today the “refereeing process works primarily to enforce orthodoxy” [8], and it is certainly true that a paper can be “refereed to death.”

But, despite its shortcomings, peer review of one sort or another has become an essential tool to help editors make decisions about the quality of manuscripts and their suitability for a particular journal. Peer review can take on different forms, and even among the smallish group of “general” journals there has never been a consensus on how best to conduct it; some journals assign just one external reviewer and then discuss the refereed paper at an internal review committee; others have three or more reviewers per manuscript, sometimes with additional methodological reviewers. For some journals, or specific manuscripts, peer review may be destined to become ever more intense. Following the suggestions of an independent committee that examined the review process for two fraudulent cloning papers, *Science* has recently announced a sort of “super review” for papers that they feel require extra scrutiny [9]. The added value of this process remains to be seen.

Whatever the specific procedure and level of intensity, journal peer review hinges on a single factor, not always obvious to authors, namely, a separation between evaluation and decision making. As explained by the entry on Wikipedia [10], “referees do not act as a group, do not communicate with each other, and typically are not aware of each other's identities. There is usually no requirement that the referees achieve consensus. Thus the group dynamics is substantially different from that of a jury.” The job of journal editors is thus clearly separate from that of reviewers; editors must assess the reviews and then come to a decision about the suitability of a particular article for *their* journal, weighing all the opinions presented. (At *PLoS Medicine*, in house editors are joined in this decision-making process by academic editors—generally members of our editorial board, but sometimes other academics—who advise us throughout the peer-review process.) As authors know to their frustration, on occasion a single review in one direction can weigh more heavily than two or more reviews in the other; editorial decisions are not made by counting beans but by taking many factors into account. The reviewers' comments are important, but so are the overall aims of the journal and other manuscripts submitted.

Subjectively, however, reviewers provide a tremendous amount of valuable advice to editors. As Callaham and Tercier say, “Most authors and editors would probably agree that the quality of peer review is crucial to selecting and publishing the best science” and, furthermore, that that peer review improves the quality of published manuscripts. A journal such as *PLoS Medicine* could not survive without its reviewers. All the above is a rather long-winded way of leading up to the main point of this editorial, which is to thank the reviewers and academic editors who have provided tremendous advice to *PLoS Medicine* in our first two years. We have listed them all at http://journals.plos.org/plosmedicine/reviewthanks.php.

Our second aim is introduce some minor changes to our system. Currently, we encourage but do not mandate open peer review, i.e., we encourage reviewers to sign their reviews, but reviewers may remain anonymous if they prefer, although we ask them to justify why. In the two years since we launched, we have found that most reviewers of research articles do not sign (in contrast, the majority of reviewers of Magazine articles do sign). When asked why they wish to remain anonymous, reviewers provide comments such as “fear”; “this author is not a forgiving person”; that they do not wish to be contacted directly by authors; and perhaps the most common: “S/he reviews my grants.” Based on our experience, most reviewers of original research are therefore not comfortable with open review. Also, we have found a bias in that reviewers are more likely to sign positive reviews. Previous trials have suggested that requiring that reviews be signed has no effect on the quality of the review [4,11], but it does make reviewers more likely to decline to review [11], as we have also found. We therefore propose to change our position on the signing of reviews; to allow but not encourage it. Reviewers will no longer be required to give reasons for not signing. However, in an attempt to make the review system more transparent in a way that we hope authors and reviewers will feel comfortable with, we will not allow reviewers to make confidential comments to editors. If a reviewer has something to say, it should be said to editors and authors together. Together with our policy not to edit reviews (except to remove typos and, very rarely, if they contain inflammatory language), authors will thus see all the comments that editors base their decision on. In addition, we will continue to share reviews between reviewers once a decision is made—a feature popular with reviewers.

So what makes a good reviewer? There are no simple answers, but a great place to start is the guidelines [12] laid out by the editor of one of the other PLoS journals, Philip Bourne. His Rule 1—Learn to say no to reviews you can't do on time—is particularly close to the hearts of most editors, but we would also stress his Rule 2: Avoid Conflict of Interest.

And to make our main point again: thank you to all our reviewers and academic editors. We are continually astounded by the insight and the time and care spent on what is a largely thankless task. Whatever the limitations of the process overall, the advice of our reviewers is crucial to us.
